# Targeting the sac or the feeders? An anatomy-driven review of contemporary embolization strategies of type II endoleaks

**DOI:** 10.1186/s42155-026-00761-0

**Published:** 2026-08-21

**Authors:** Maciej Błaszyk, Jakub Waliszewski, Zuzanna Fryska, Dominika Gargula, Patryk Wruck, Kinga Karbowiak, Robert Juszkat

**Affiliations:** Department of Clinical Radiology, Poznan University of Medical Sciences, Poznań, Poland

## Abstract

Endovascular aortic repair (EVAR) has become the primary treatment for abdominal aortic aneurysms; however, type II endoleaks (T2ELs) remain the most frequent post-procedural complication, with variable clinical significance and management strategies. This review synthesizes current evidence on the natural history, diagnostic evaluation, and treatment of T2EL, with a focus on the comparative roles of aneurysm sac and feeder embolization. Available data indicate that T2ELs represent a heterogeneous condition that often follows a benign course with a substantial rate of spontaneous resolution, whereas a clinically significant subset is associated with aneurysm sac enlargement requiring intervention. Across studies, treatment outcomes appear to be driven primarily by anatomical complexity and flow patterns rather than by the choice of embolization technique alone. Transarterial embolization remains the most commonly used first-line approach, whereas direct sac puncture has demonstrated higher technical and clinical success rates in selected complex cases. However, direct comparison between strategies is limited by heterogeneity in outcome definitions, study design, and follow-up protocols. Nevertheless, we present an anatomy-driven decision algorithm for selecting the embolization method based on the T2EL complexity. These findings support a shift from technique-centered to anatomy-driven therapeutic decision-making. In selected cases with limited inflow, feeder embolization may be sufficient, whereas complex endoleaks with multiple collateral pathways often require direct or indirect sac embolization to achieve durable exclusion. An individualized, anatomy-based treatment strategy appears to provide the most rational framework for optimizing outcomes in patients with T2EL.

## Introduction

Endovascular aortic repair (EVAR) has become the predominant treatment modality for abdominal aortic aneurysms (AAAs) [1]. Compared with open aneurysm repair (OAR), EVAR is associated with reduced blood loss, shorter hospital stays, and lower rates of systemic complications [2]. However, endoleaks remain a common complication, occurring in approximately 10–40% of cases, and are defined as persistent blood flow within the aneurysm sac following stent-graft placement [3].

Endoleaks are classified into five types: type I (attachment-site endoleak; Ia—proximal, Ib—distal), type II (retrograde perfusion of the aneurysm sac via branch vessels), type III (graft defect), type IV (graft porosity), and type V (endotension) [4]. Among these, type II endoleaks (T2ELs) are the most common subtype, accounting for approximately half of all endoleaks. Notably, up to one-third to one-half of T2ELs resolve spontaneously without intervention [5, 6].

Current recommendations for intervention include persistent T2EL associated with significant aneurysm sac enlargement (≥ 10 mm) or the development of related complications, such as sac rupture, after exclusion of type I or III endoleaks [7, 8].

Two principal treatment strategies are currently used: embolization of the aneurysm sac and embolization of the feeding vessels. Despite the widespread clinical use of both approaches, there is no clear consensus on their relative efficacy, largely because of heterogeneity in the available literature and differences in patient selection, anatomical complexity, and technical execution [9, 10].

This review provides a clinically oriented comparison of sac and feeder embolization strategies for T2EL, with particular emphasis on technical aspects, indications, and their impact on clinical outcomes.

Existing reviews have primarily organized treatment according to access route or embolization technique, while providing limited practical guidance on how the therapeutic target should be selected according to endoleak architecture and hemodynamic complexity. The present review therefore reframes treatment selection around the primary target—feeding vessels, the endoleak cavity, or both—and translates this concept into a clinical decision algorithm.

## Review methodology

This clinically oriented narrative review summarizes current evidence on the pathophysiology, imaging assessment, and endovascular management of T2EL following EVAR, with particular emphasis on selection of the embolization target. PubMed/MEDLINE was searched for English-language, full-text publications from January 2000 through July 2026 using Medical Subject Headings and free-text terms related to type II endoleak, EVAR, transarterial embolization, direct sac puncture, and translumbar embolization. Reference lists of relevant articles and current guidance documents were also screened. The search was last updated on 30th July 2026, and study selection was performed by two reviewers.

Eligible publications addressed adult patients with T2EL after EVAR and reported information on natural history, imaging, indications for intervention, embolization strategies, procedural outcomes, sac behavior, recurrence, or complications. When multiple publications addressed the same clinical question, priority was given to evidence-based clinical practice guidelines, systematic reviews and meta-analyses, prospective or multicenter comparative studies, larger retrospective cohorts, and finally, smaller series or technical reports. Standards-of-practice documents and expert reviews were used primarily to provide technical and procedural context. Owing to substantial clinical and methodological heterogeneity, no quantitative synthesis was undertaken, and the evidence was assessed qualitatively.

## Natural history of T2EL—indications for treatment

Although the prevalence of T2EL is well established, reported incidence varies widely across studies, ranging from 10 to 34% [6, 10]. In the majority of cases, T2EL follows a benign clinical course and does not require intervention. A watchful waiting strategy is therefore commonly adopted, with spontaneous resolution reported in approximately 35–54% of cases [6, 11].

Several studies support the generally favorable natural history of T2ELs. In a cohort reported by Steinmetz et al., 38.9% of patients had persistent T2EL; however, aneurysm sac enlargement occurred in only a small subset of these cases [12]. Importantly, the risk of aneurysm rupture in patients with isolated T2EL is low, estimated at less than 1% [13]. Similarly, Sidloff et al. reported no cases of aneurysm rupture in a cohort of 175 patients with T2EL [11]. Although the risk of this complication is relatively low, the overall mortality rate in cases of aneurysm rupture, not just in case of T2EL, is approximately 81% [14]. Furthermore, current evidence suggests that T2EL is not associated with reduced overall survival [15].

According to the 2024 Cardiovascular and Interventional Radiological Society of Europe (CIRSE) standards, computed tomography angiography (CTA) is recommended at 1 month following EVAR. In cases of confirmed T2EL, after exclusion of type I and III endoleaks and in the absence of sac expansion, follow-up imaging with CTA at 12 months is advised [13].

However, European Society of Vascular Surgery (ESVS) in 2024 guidelines recommend to consider secondary intervention in the presence of aneurysm sac growth ≥ 10 mm compared with baseline or with the smallest diameter during follow-up using the same imaging modality and measurement method, whereas stable sacs should be monitored [8].

## Pathophysiology

Experimental models suggest that T2EL is driven by pressure gradients between the aneurysm sac and feeding arteries; the relatively higher pressure within the inferior mesenteric artery (IMA) may explain its frequent role as a dominant inflow vessel [16].

**Fig. 1:**
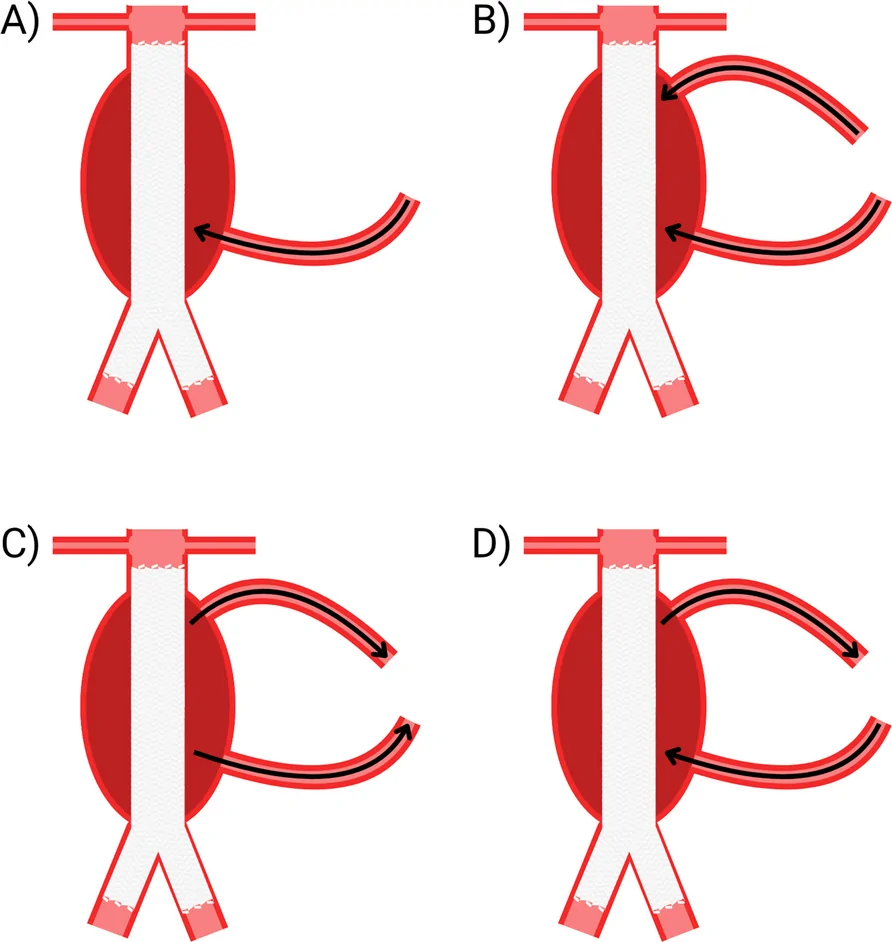
Subtypes of type II endoleaks: **a** type IIa endoleak—single vessel, **b** type IIb endoleak—multiple vessels forming a collateral network (blood inflow), **c** type IIb endoleak—multiple vessels forming a collateral network (blood outflow), **d** type IIb endoleak—multiple vessels forming a collateral network (blood flow through the sac)

Two main subtypes of T2EL can be distinguished by the vascular pattern of retrograde flow into the aneurysm sac. Type IIa endoleaks are characterized by a single feeding vessel, whereas type IIb endoleaks involve multiple vessels that form a complex collateral network. These vessels communicate through the aneurysm sac, thereby creating both inflow and outflow pathways. Subtypes of T2EL are presented in Fig. 1. Clinically, type IIa endoleaks are more likely to resolve spontaneously, whereas type IIb endoleaks are more frequently associated with persistence and aneurysm sac enlargement [17–20].

Type IIa endoleaks most commonly arise from a patent IMA or lumbar arteries (LAs). Less common sources include accessory renal, gonadal, median sacral, and internal iliac arteries. Differentiating between type IIa and type IIb endoleaks can be challenging with standard CTA, which may limit accurate characterization of inflow and outflow patterns. Advanced imaging techniques, including dynamic or time-resolved CTA and four-dimensional flow-sensitive magnetic resonance imaging, may improve characterization of endoleak architecture and hemodynamics in cases where standard CTA is insufficient and more dynamic imaging is needed [21, 22].

The role of pre-emptive IMA embolization prior to EVAR remains a subject of controversy. Although earlier studies proposed a potential reduction in the incidence of T2EL, more recent evidence does not substantiate a significant benefit. Specifically, randomized data have demonstrated no significant differences in aneurysm sac volume, sac diameter change, T2EL occurrence, reintervention rates, or overall survival at follow-up [23]. These findings suggest that routine prophylactic embolization of the IMA is not justified.

Various anatomical and clinical factors influence the development and persistence of T2EL. Recognized risk factors encompass advanced age, patent IMA, larger aneurysm diameter, and a greater number of patent LAs [18, 24, 25]. Persistent T2EL has also been associated with prior embolization of hypogastric arteries and distal graft extension. Conversely, smoking has shown an inverse association with T2EL occurrence and chronic obstructive pulmonary disease (COPD) has been identified as a potential protective factor. This association may reflect smoking-related vascular changes and a greater propensity for intraluminal thrombus formation within the aneurysm sac. Increased thrombus burden may reduce the patency of potential feeding vessels and thereby lower the risk of T2EL [26–29]. Additionally, the presence of fewer than six patent LAs has been correlated with an increased likelihood of spontaneous aneurysm sac reduction [30].

Several predictive models have been introduced to stratify the risk of T2EL occurrence and persistence. For instance, Mo et al. classified patients into high-, intermediate-, and low-risk categories based on IMA diameter and the number of patent LAs [31]. Although these models offer valuable insights, their clinical utility remains constrained by differences in study design and outcome definitions. However, they identify a subgroup of patients undergoing EVAR who may be at increased risk of T2EL and therefore require closer surveillance. Several researchers have identified risk factors for T2EL incidence, which are summarized in Table 1.

**Table 1:** Factors associated with T2EL occurrence, persistence, sac expansion, or reintervention

Study, publication year	Outcomes
Kondov et al. [32] 2022	**Increased risk of T2EL with:** - (IMA diameter + number of patent LAs)/2 > 3.9
Liu et al. [25] 2023	**Increased risk of T2EL with:** - IMA diameter > 2.77 mm - < 45.5% of thrombus volume in aneurysm sac
Li et al. [33] 2019	**Increased risk of T2EL-related reintervention with:** - Lower thrombus volume - Higher number of patent LAs - Higher number of infrarenal aortic branch vessels - Higher summed diameters of patent infrarenal aortic branch vessels
Ward et al. [34] 2014	**Increased risk of T2EL with:** - Patent IMA - Higher number of patent LAs
Nicholls et al. [29] 2022	**Increased risk of persistent T2EL or reintervention with:** - Patent IMA - Low thrombosis burden (TB)^1^
Dudeck et al. [35] 2015	**Increased risk of reintervention, based on patients with T2EL on CTA:** - Higher EL volume - Higher EL diameter - Higher number of aortic branch vessels - % of “complex type” endoleak pattern
Sugimoto et al. [36] (2026)	**Increased risk of sac expansion:** - High pre-operative D-dimer - High pre-operative D-dimer and high intraluminal thrombus ratio (D-dimer was not predictive in the low intraluminal thrombus ratio)

^1^Thrombosis burden (TB) is the difference of aortic diameter and flow lumen diameter, divided by aortic diameter; low TB < 25%, medium TB 25–50%, high TB > 50%

*T2EL* type II endoleak, *IMA* inferior mesenteric artery, *LA* lumbar artery, *TB* thrombosis burden, *CTA* computed tomography angiography

These anatomical and hemodynamic differences directly influence whether embolization should target the feeding vessels, the aneurysm sac, or both [37].

## Imaging for treatment planning

Imaging in patients with T2EL should serve not only to detect the endoleak but also to determine whether intervention is warranted and to define the therapeutic target. According to the 2024 ESVS Clinical Practice Guidelines, longitudinal assessment should focus on aneurysm sac behavior using a consistent imaging modality and measurement method [8].

Post-EVAR surveillance requires systematic imaging follow-up, with CTA performed at approximately 30 days after the procedure to detect potential endoleaks. Once an endoleak is identified, accurate classification is essential, particularly to confirm T2EL, which accounts for more than half of all endoleaks following EVAR [5, 6].

In the absence of an endoleak, repeat CTA should be performed after 5 years. For patients with T2EL, annual follow-up with Doppler ultrasound (DUS) or CTA is recommended to monitor sac morphology, persistence of endoleak, and feeding vessel patency ESVS puts crucial emphasis on sac dynamic in long term follow-up. Favorable sac dynamic is indication for less intensive follow-up diagnostics (from annual CTA or DUS to CTA in 5 years), whereas unfavorable sac dynamic should be evaluated for reintervention [8, 38–40].

CTA remains the reference standard for post-EVAR imaging because of its high spatial resolution and comprehensive assessment of aneurysm morphology. However, contrast-enhanced ultrasound (CEUS) has demonstrated comparable sensitivity for detecting T2EL and offers several advantages, including real-time assessment of inflow and outflow vessels, evaluation of flow direction and velocity, and avoidance of ionizing radiation [38, 41, 42]. Its limitations include operator dependence and reduced diagnostic accuracy in patients with obesity or excessive bowel gas [38, 43].

Magnetic resonance angiography (MRA) constitutes an alternative imaging modality characterized by high sensitivity for T2EL detection and superior soft tissue contrast. Nonetheless, its application is constrained by factors such as availability, cost, extended acquisition durations, and susceptibility to artifacts associated with specific stent-graft materials [38].

Regardless of the imaging modality used, the primary objective of imaging evaluation is to guide treatment planning. Imaging should provide detailed information on aneurysm sac morphology, the number and type of feeding vessels, their anatomical course, and potential access routes for embolization, all of which are critical for selecting the optimal therapeutic strategy.

## Single feeder

Contemporary management of T2EL increasingly focuses on achieving complete exclusion of the endoleak cavity, often through combined embolization of both the feeding vessels and the aneurysm sac [44, 45]. As a result, isolated embolization of a single feeding artery is performed less frequently and is generally reserved for highly selected cases, such as well-defined type IIa endoleaks arising from a dominant IMA or a single LA [46].

The most prevalent method employed is transarterial access, usually via the common femoral artery, which facilitates catheterization of both the feeding vessel and, in certain cases, the aneurysm sac [28]. Access to the target vessel is typically achieved through collateral pathways. Catheterization of the IMA is generally more feasible owing to its relatively larger diameter, despite its lengthy, tortuous course, whereas access to LAs often poses greater technical difficulty due to their smaller caliber and intricate communication with the iliolumbar arterial network [13].

Prophylactic IMA embolization is not discussed here as a treatment strategy for established T2EL, and randomized data do not currently support its routine use for prevention [23].

Importantly, embolization of a feeding vessel alone is often insufficient to achieve durable endoleak exclusion. When the aneurysm sac is not accessed and embolized, persistent flow may continue through collateral pathways, leading to treatment failure [6]. Therefore, when access to the aneurysm sac cannot be achieved, alternative treatment strategies should be considered, with priority given to approaches that enable direct or indirect embolization of the aneurysm sac.

## Collateral feeders and sac embolization

In cases with multiple feeding vessels, poorly defined inflow and outflow pathways, or a large, persistent endoleak cavity, sac-directed embolization should be strongly considered. Direct targeting of the endoleak cavity may enable complete disruption of the hemodynamic circuit, particularly for complex T2EL, in which selective feeder embolization alone is unlikely to be sufficient [13, 28, 47].

Two principal minimally invasive sac-targeting strategies are currently used: transarterial embolization and direct sac puncture (DSP) [13].

### Transarterial approach

Transarterial embolization remains the most commonly used first-line approach for managing T2EL. The objective of this technique is to advance the microcatheter as close as possible to the aneurysm sac and to embolize both the feeding vessel and, when feasible, the endoleak nidus. Access is typically obtained via transfemoral or transbrachial routes, most commonly targeting the IMA or LAs [13, 45, 46, 48].

Interpretation of clinical outcomes is complicated by heterogeneity in the definition of clinical success, which has been variably reported as the absence of sac enlargement, a reduction in sac diameter, or the complete absence of flow on follow-up imaging [47]. In contrast, technical success is consistently defined as the successful delivery of embolic material into the endoleak cavity [49].

Reported technical success rates for transarterial embolization range from 71 to 87%, indicating that the procedure is feasible in the majority of cases [50, 51]. However, clinical success rates are more variable. For example, Moosavi et al. reported sac stability in only 33% of patients, whereas DSP achieved a clinical success rate of 75%. However, longer follow-up in the transarterial group may have contributed to these differences [51]. Other series, such as that by Müller-Wille et al., reported clinical success rates of approximately 73%, defined as stability or reduction in aneurysm sac volume [48].

Variability in reported outcomes likely reflects differences in embolic materials, procedural endpoints, operator experience, and the degree to which the endoleak cavity rather than only the proximal feeder is embolized. Mechanisms of treatment failure include persistent collateral perfusion, recanalization, and the development of new feeding pathways [6].

### Target vessels: IMA and LAs

Transarterial embolization via the IMA is the most commonly performed approach, typically accessed through the superior mesenteric artery. Embolic agents include coils, liquid embolics, or a combination of both [13].

Conversely, embolization of lumbar arteries presents greater technical difficulty owing to their smaller caliber and intricate communication with the iliolumbar arterial network. In certain instances, catheterization of these vessels may prove anatomically unfeasible. Alternative methods, such as the injection of low-viscosity Onyx through the iliolumbar artery, have been documented as facilitating indirect access to the endoleak nidus in selected cases [45].

**Fig. 2:**
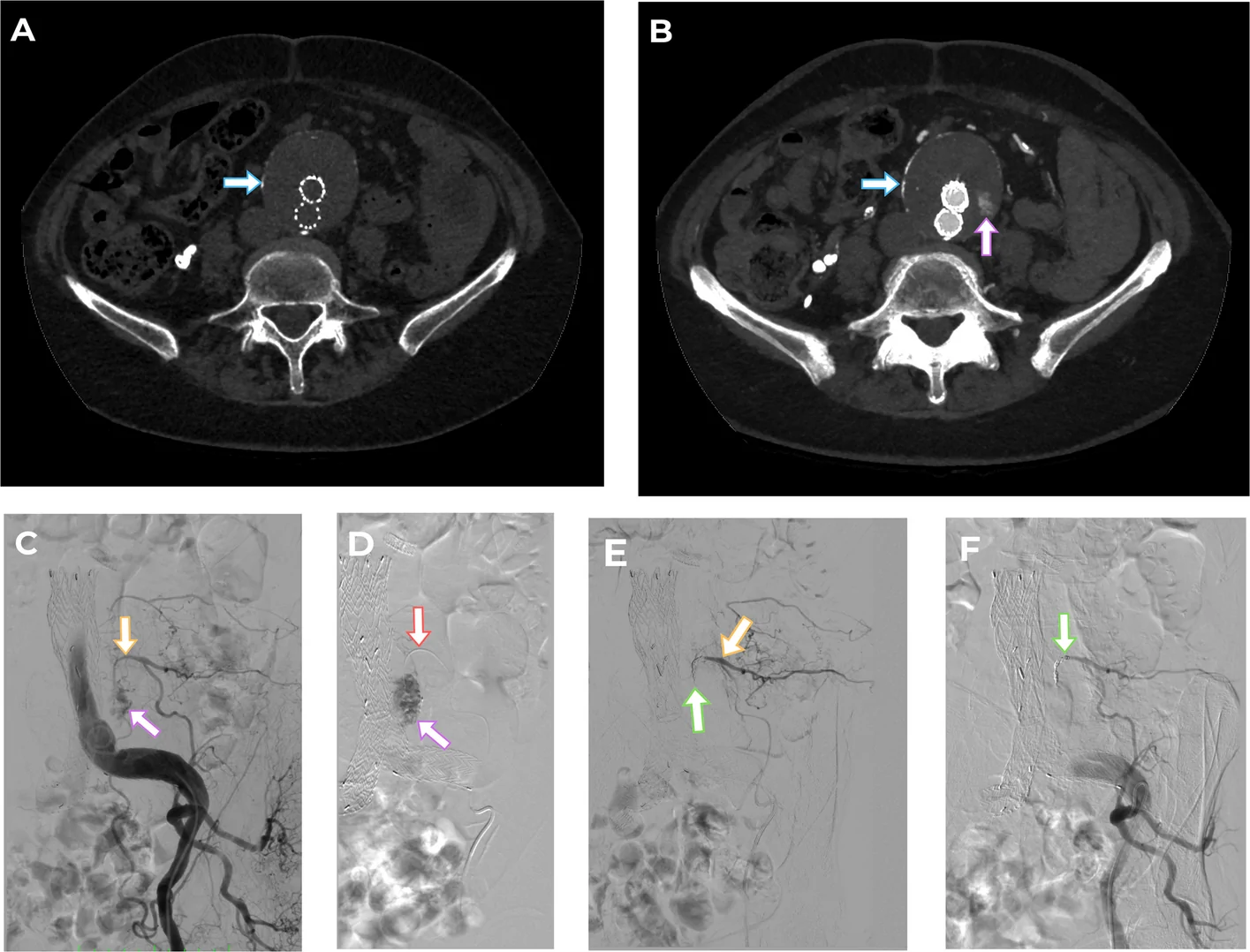
Imaging findings and procedural steps of transarterial coil embolization of a type II endoleak feeding vessel. **A** Unenhanced axial CT shows the aneurysm sac surrounding the endograft (blue arrow). **B** Contrast-enhanced CTA demonstrates the type II endoleak cavity (pink arrow) within the aneurysm sac (blue arrow). **C** DSA performed through a pigtail catheter demonstrates the feeding vessel (orange arrow) and opacification of the endoleak cavity (pink arrow). **D** Selective microcatheter DSA (red arrow) confirms filling of the endoleak cavity (pink arrow). **E** Selective angiography after coil (EV3 Concerto) deployment shows the coil pack within the treated feeding vessel (green arrow) and no residual opacification of the endoleak cavity; the feeding artery is indicated by the orange arrow. **F** Completion digital subtraction angiography confirms stable coil position (green arrow) and absence of residual endoleak filling. This example represents an anatomically simple endoleak with an accessible dominant feeder; immediate angiographic occlusion should not be considered equivalent to durable clinical success

Imaging findings and procedural steps of transarterial coil embolization of a type II endoleak feeding vessel are shown in Fig. 2.

### DSP

DSP represents an alternative sac-targeting technique, performed via percutaneous access to the aneurysm sac. Two principal approaches are used: transabdominal (supine position) and translumbar (prone position). Under ultrasound or CT guidance, a needle is advanced into the aneurysm sac along a predefined safe trajectory based on prior imaging [13]. Structures that must be carefully avoided include the inferior epigastric artery, mesenteric arteries, and bowel. During the procedure, constant pressure should be maintained during needle advancement to displace the bowel away from the puncture trajectory [52].

**Fig. 3:**
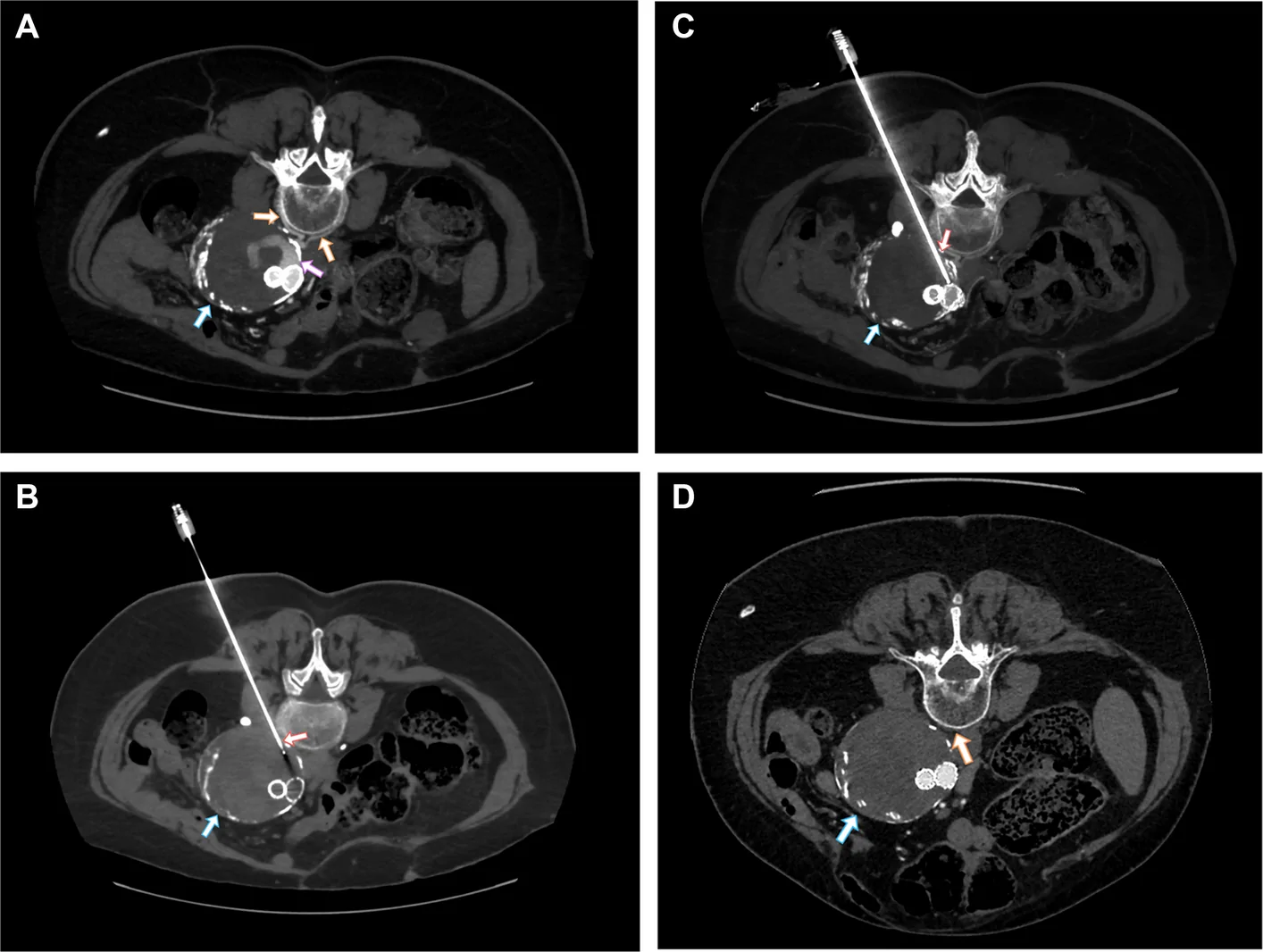
Imaging findings and procedural steps of CT-guided direct sac puncture embolization of a type II endoleak. **A** Contrast-enhanced CTA localizes the endoleak cavity (pink arrow) within the aneurysm sac (blue arrow) and lumbar arteries as feeding vessels (orange arrows) and facilitates planning of the percutaneous access route. **B** Intraprocedural CT confirms positioning of the needle tip (red arrow) within the endoleak cavity; the aneurysm sac is indicated by the blue arrow. **C** Immediate post-embolization contrast-enhanced CTA after injecting 4 mL of thrombin confirms the needle position (red arrow) and demonstrates no residual enhancement; the aneurysm sac is indicated by the blue arrow. **D** Eight-month follow-up contrast-enhanced CTA demonstrates stable aneurysm sac size (blue arrow) and no recurrent type II endoleak, orange arrow shows previous feeding vessel without contrast enhancement

Imaging findings and procedural steps of CT-guided direct sac puncture embolization of a type II endoleak are shown in Fig. 3.

The embolic materials used are similar to those used in transarterial techniques [52]. Both technical and clinical success rates have been reported to be higher with DSP than with transarterial embolization [6, 49]. Technical success may reach up to 97%, while clinical success rates range from approximately 78% to 81% [6, 52]. DSP may be associated with fewer complications than transarterial embolization [6].

However, these comparisons should be interpreted cautiously because of heterogeneity in patient selection, definitions of clinical success, follow-up duration, and the frequent use of DSP in anatomically complex or refractory cases. Despite these advantages, transarterial embolization remains the preferred first-line approach in many centers. This preference largely reflects its less invasive nature, avoidance of DSP, and broader operator familiarity. In contrast, DSP is often reserved for anatomically complex or refractory cases, particularly when transarterial access to the endoleak cavity is not feasible.

### Adjunctive techniques

When standard embolization techniques prove unsuccessful, alternative approaches such as transiliac paraendograft or transcaval embolization should be considered. The transiliac paraendograft approach entails accessing the endoleak cavity via the potential space between the iliac limb of the endograft and the arterial wall. The transcaval technique, which involves percutaneous puncture of the inferior vena cava, may be particularly advantageous when the endoleak cavity is situated on the right side of the aneurysm sac [13].

Open surgical repair remains the most definitive treatment option, with reported technical success rates approaching 100% and excellent control of aneurysm sac enlargement. However, due to its invasiveness, it is typically reserved for cases in which endovascular approaches have failed [49].

## Embolic agents

Embolic materials used for the treatment of T2EL include coils and liquid embolic agents.

Coils are primarily used for selective embolization of larger feeding vessels, such as aortic side branches. Several studies have suggested that coil embolization alone may be associated with higher reintervention rates than treatment with liquid embolic agents [34, 53].

However, these findings should be interpreted cautiously because studies evaluating coils generally have longer follow-up periods than those assessing liquid embolic materials. Although Kim et al. reported no significant differences between coil embolization and Onyx embolization, the considerably shorter follow-up period in the Onyx group limits the comparability of the outcomes and should be taken into account when interpreting these findings [37, 54].

Liquid embolic agents include ethylene–vinyl alcohol copolymer (Onyx) and N-butyl cyanoacrylate (NBCA). These agents solidify upon injection following contact with blood, Onyx contains ethylene–vinyl alcohol copolymer dissolved in dimethyl sulfoxide (DMSO). DMSO diffuses into the surrounding aqueous environment, the copolymer precipitates and forms a cohesive embolic cast.

NBCA is composed of monomers that polymerize upon contact with blood, forming a solid material. Liquid embolization is typically used when feeding vessels are small or difficult to catheterize. The embolic agent may spread from the injection site through the endoleak cavity and reach outflow branches without selective catheterization of each vessel [37, 54, 55].

A major disadvantage of Onyx is its relatively high cost, which is substantially greater than that of coils. In addition, no specific guidelines define the maximum safe volume of Onyx that can be administered [54].

NBCA must be mixed with ethiodized oil to provide radiopacity. Its polymerization time depends on the NBCA-to-oil dilution ratio [55].

Other agents, including thrombin and gelatin-based materials, have been used during direct sac puncture in selected cases [51].

Different embolic materials may also be used in combination to optimize treatment effectiveness. However, no guidelines currently define the optimal embolic agent for T2EL treatment. Therefore, the embolization strategy should be individualized according to CTA findings, endoleak anatomy, and operator experience [37].

## Fluoroscopy time

The choice of therapeutic approach for T2EL embolization is determined not only by vascular access and aneurysm sac anatomy or physiology but also by procedural considerations. In an individualized treatment strategy, fluoroscopy time may be an important factor. However, fluoroscopy time should not be considered equivalent to the total radiation dose delivered to the patient during the procedure.

Yang et al. reported a substantially shorter mean fluoroscopy time for DSP than for the transarterial approach, at 11.2 and 42.4 min (*p* < 0.001), respectively. The mean procedure time was also shorter with DSP, at 103 min compared with 181 min for the transarterial approach (*p* < 0.001) [56].

Zener et al. reported similar results for DSP, although without direct comparison with the transarterial approach. The median fluoroscopy time was 11.3 min, and the median procedure time was 90 min [52].

Fanelli et al. reported a slightly longer mean fluoroscopy time of 16.7 min but a considerably shorter mean procedure time of 51.36 min [57].

## Discussion

Despite the considerable prevalence of T2EL, their clinical significance and ideal management strategies continue to be topics of ongoing discussion [6, 10, 20]. While numerous T2ELs tend to follow a benign progression, a subset is linked with aneurysm sac enlargement and necessitates intervention [11, 13]. The variability observed in reported outcomes across studies underscores the heterogeneous nature of T2EL, especially concerning anatomical configuration and flow dynamics [17, 19, 20].

**Fig. 4:**
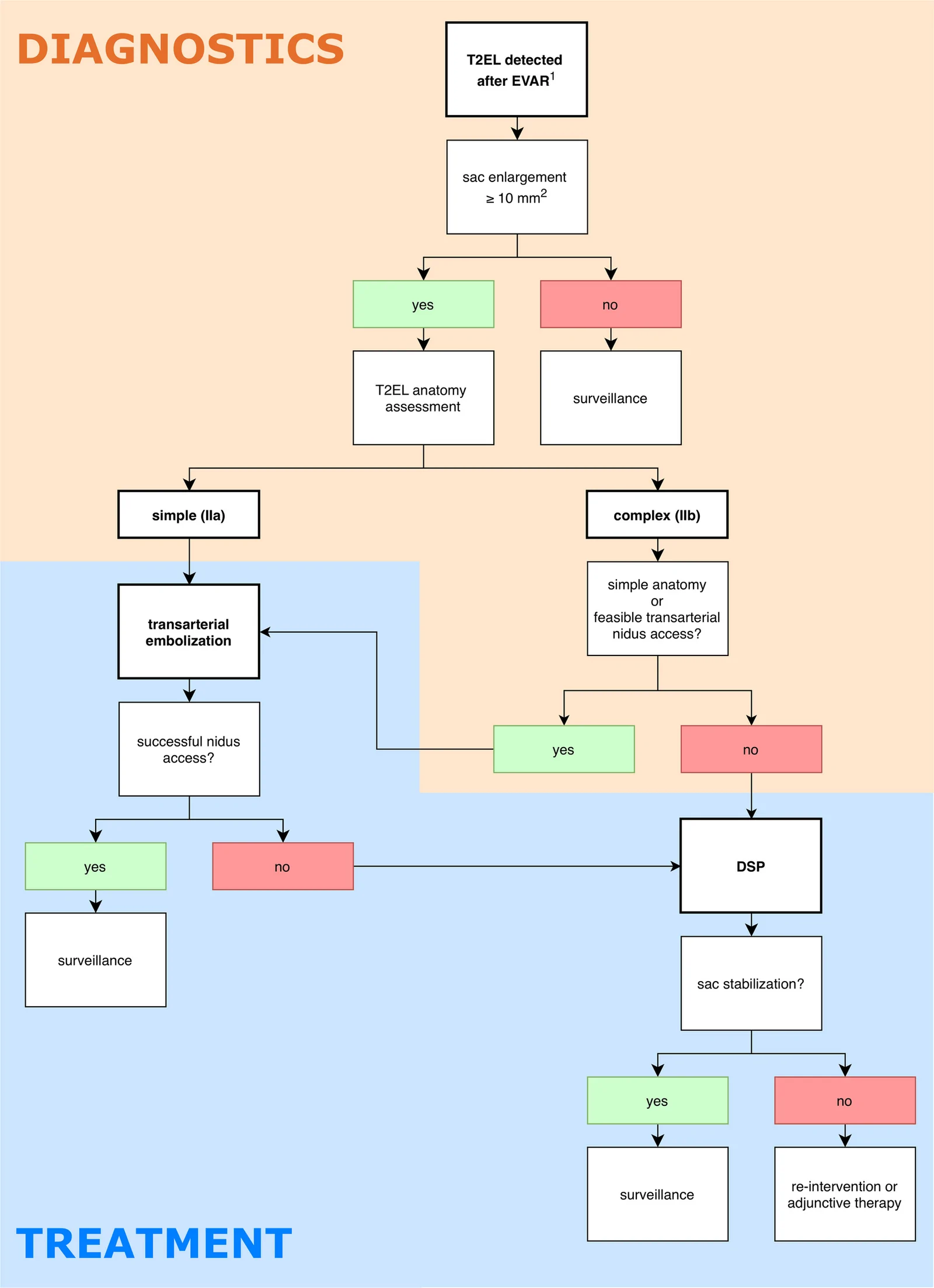
Anatomy-driven decision algorithm for selecting the embolization method based on the T2EL complexity. ^1^After exclusion of type I/III endoleak or other cause of sac enlargement; ^2^compared with baseline or with the smallest diameter during follow-up using the same imaging modality and measurement method. T2EL, type II endoleak; DSP, direct sac puncture

Feeder- and sac-directed embolization should be regarded as complementary strategies selected according to anatomy rather than as competing techniques. Selective feeder embolization is reasonable for type IIa and anatomically simple type IIb endoleaks when the dominant inflow or outflow pathway and the nidus can be reached. Complex type IIb endoleaks—with multiple or uncertain channels, recurrent perfusion, or network complexity underestimated on pre-procedural imaging—are better treated as sac-centered hemodynamic circuits using transarterial nidus embolization, DSP, or another sac-directed route. Because imaging may not reliably distinguish type IIa from type IIb anatomy, transarterial embolization remains a pragmatic first-line approach when nidus access is feasible; the proposed algorithm formalizes this anatomy-driven selection (Fig. 4) [13, 21, 37, 46, 51].

The substantial difference in fluoroscopy time between DSP and the transarterial approach, favoring DSP, may be attributable to fundamental differences between the two techniques and their respective endovascular requirements. This hypothesis is supported by the considerable difference in total procedure time observed between the approaches. However, the available literature on this topic remains limited, and further comparative studies are required to confirm this association and identify its underlying determinants [52, 56, 57].

The apparent superiority of DSP over transarterial embolization in some studies should be interpreted cautiously [6, 49, 51]. Differences in follow-up duration, definitions of clinical success, and patient selection can substantially affect reported outcomes [47]. In particular, DSP is often used for more complex or refractory cases, which may introduce selection bias. At the same time, transarterial embolization remains widely used as a first-line approach because of its less invasive nature and broader applicability, especially in anatomically favorable scenarios where catheter-based access to the feeding vessel and endoleak cavity is achievable [13, 45, 46, 48].

Diagnostic imaging plays a crucial role in treatment planning. Although conventional CTA remains the gold standard for post-EVAR surveillance and diagnosis, alternative modalities such as MRA and CEUS may provide more detailed and dynamic information on aneurysm sac anatomy and behavior, despite their respective limitations [38, 41–43].

Comparable intraoperative information may be obtained using cone-beam computed tomography (CBCT), with the potential additional benefit of reducing contrast-agent use. CBCT is a valuable tool for intraoperative image acquisition, endovascular navigation, localization of catheters and guidewires, and, most importantly, precise targeting of the treatment site during both transarterial and direct sac puncture procedures [58].

The heterogeneity of the available literature further complicates direct comparisons between treatment strategies [10, 47]. Clinical success has been variably defined as the absence of sac enlargement, sac reduction, or complete resolution of flow, and follow-up protocols differ substantially between studies [47]. Additionally, variations in embolic materials, operator experience, and procedural techniques contribute to the broad spectrum of reported outcomes [6, 45, 48]. These factors likely account for much of the inconsistency observed in the literature, rather than genuine differences in efficacy among treatment modalities.

From a practical standpoint, these findings support an individualized, anatomy-driven approach to T2EL management [10, 47]. Detailed pre-procedural imaging is essential for characterizing the number and type of feeding vessels, the presence of collateral networks, and the accessibility of the aneurysm sac [13]. In selected cases with limited inflow, feeder embolization may be sufficient [46]. However, in more complex anatomical settings, strategies targeting the aneurysm sac—either via transarterial access or via DSP—are more likely to achieve sustained endoleak exclusion [13, 28, 47].

This review has several limitations. Its narrative design and reliance on heterogeneous studies preclude direct quantitative comparisons of treatment strategies [10, 47]. Nevertheless, it reflects current clinical practice and highlights key factors that influence treatment selection.

## Conclusions

T2ELs represent a heterogeneous group of post-EVAR complications, characterized by variable clinical significance and outcomes. Although many cases exhibit a benign course and can be monitored safely, a subset necessitates intervention, especially in instances of aneurysm sac enlargement.

Current evidence does not support the superiority of any single embolization technique. Instead, treatment outcomes appear to be primarily determined by underlying anatomical complexity and flow patterns. In simple configurations, selective feeder embolization may be sufficient. In contrast, complex endoleaks with multiple collateral pathways often require direct or indirect sac embolization to achieve durable exclusion of the endoleak circuit.

Although DSP has demonstrated higher reported success rates in some series, transarterial embolization remains the most widely adopted first-line approach due to its lower invasiveness and broader applicability. In practice, these strategies should be considered complementary rather than competitive.

An individualized, anatomy-driven approach, based on detailed pre-procedural imaging and careful assessment of vascular architecture, appears to provide the most rational framework for optimizing outcomes in patients with T2ELs.

## Data Availability

Not applicable.

## Abbreviations

AAA: Abdominal aortic aneurysm
CDUS: Color Doppler ultrasound
CEUS: Contrast-enhanced ultrasound
CIRSE: Cardiovascular and Interventional Radiological Society of Europe
COPD: Chronic obstructive pulmonary disease
CTA: Computed tomography angiography
DMSO: Dimethyl sulfoxide
DSP: Direct sac puncture
DUS: Doppler ultrasound
EVAR: Endovascular aneurysm repair
ESVS: European Society of Vascular Surgery
IMA: Inferior mesenteric artery
LA: Lumbar arteries
MRA: Magnetic resonance angiography
MRI: Magnetic resonance imaging
NBCA: N-butyl cyanoacrylate
OAR: Open aneurysm repair
T2EL: Type II endoleak
